# Histone citrullination: a new target for tumors

**DOI:** 10.1186/s12943-021-01373-z

**Published:** 2021-06-11

**Authors:** Dongwei Zhu, Yue Zhang, Shengjun Wang

**Affiliations:** 1 Department of Laboratory Medicine, The Affiliated People’s Hospital, Jiangsu University, Zhenjiang, 212013 China; 2 Department of Immunology, Jiangsu Key Laboratory of Laboratory Medicine, School of Medicine, Jiangsu University, Zhenjiang, China

**Keywords:** Histone, Citrullination, Tumor, Protein arginine deiminases, NET

## Abstract

As the main protein components of chromatin, histones play central roles in gene regulation as spools of winding DNA. Histones are subject to various modifications, including phosphorylation, acetylation, glycosylation, methylation, ubiquitination and citrullination, which affect gene transcription. Histone citrullination, a posttranscriptional modification catalyzed by peptidyl arginine deiminase (PAD) enzymes, is involved in human carcinogenesis. In this study, we highlighted the functions of histone citrullination in physiological regulation and tumors. Additionally, because histone citrullination involves forming neutrophil extracellular traps (NETs), the relationship between NETs and tumors was illustrated. Finally, the clinical application of histone citrullination and PAD inhibitors was discussed.

## Background

Nucleosomes are the fundamental units of chromatin, comprising central histones and packaged DNA [1–3]. Most of the four core histone proteins (H2A, H2B, H3, and H4) consist of an α-helical C-terminal domain that allows histone-histone interactions to form the octameric column-like structure onto which the DNA is wrapped [4]. The remaining 25% of the core histones consists of the largely structurally undefined but evolutionarily conserved “tail” domains. The “tail” domains are readily accessible to enzymes that perform important posttranslational modifications (PTMs) for epigenetic regulation. The citrullination of linker histone (H1) was also identified as a regulator of chromatin decondensation [5]. Compared with canonical histone, histone variants can affect nucleosome stability and help create functionally distinct chromatin domains. A recent study identified the first *Drosophila* H1 histone variant, called dBigH1, which regulates zygotic genome activation [6]. Following DNA damage, the H2A variant H2A.X can be phosphorylated at serine 139, facilitating recruitment of the DNA damage repair machinery [7]. H2A.B appeared late in evolution in mice and humans, and RNA Pol II might be recruited to splicing speckles by H2A.B to facilitate high transcription levels [8]. A particular mammalian H3 variant, called cenH3, replaces H3 at centromeric regions and is important for centromere propagation and maintenance [9]. Additionally, H3.3 (H3 variant) incorporation induces an open chromatin conformation and increases transcription by damaging high-order chromatin formation [10]. Furthermore, the phosphorylation of the histone H2A variant (H2AX) participates in the DNA damage response and DNA double-strand breaks [11]. Given the close relationship between histone and DNA, histone modifications have considerable impacts on DNA-template processes, such as transcription, replication, repair and recombination [12, 13]. Certain covalent PTMs can modify the charge density of histones and DNA, affecting the structure of chromatin and transcription process. However, modifications can also change the structure or function of chromatin by recognizing specific binding proteins [14]. The PTMs of histones are associated with many diseases, highlighting the importance of the mechanisms and functions of histone modifications [15, 16].

Citrullination is a PTM catalyzed by the peptidyl arginine deiminase (PAD) enzyme family [17], which includes 5 isoenzymes (PAD1–4 and PAD6) with various tissue-specific targets. This process depends on an increased calcium concentration [18]. Under physiological conditions, the activity of the PAD enzyme is low. However, under pathological conditions, PAD enzymes can citrullinate many structural proteins, including vimentin, keratin and filaggrin [19, 20], and some histones such as H1, H2A, H3 and H4 [5, 21, 22]. Further studies have demonstrated the role of histone citrullination in autoimmune diseases. For example, the most specific antibody to rheumatoid arthritis (RA) is the antibody against citrullinated protein (ACPA) [23], which can be detected early, reflects the outcome of RA and serves as a useful diagnostic and prognostic tool for RA [24]. More than 90% of RA patients have anti-citrullinated H2B antibodies caused by respiratory infections [25]. The immune complexes containing citrullinated histones can activate the cytokine production of macrophages and proliferation of neutrophils. Several lines of evidence that link neutrophil extracellular traps (NETs) to RA, such as the sera from RA patients with Felty’s syndrome, can bind to CitH3 and NETs or CitH4 contained in NETs. Additionally, human monoclonal antibodies produced by RA synovial B cells decorate NETs and bind to the citrullinated histones [26]. PAD4 may contribute to the pathogenesis of RA by inducing NETs through citrullinated histones. Consistent with this theory, PAD4-deficient mice reduce the formation of NETs, autoantibodies, and arthritis, indicating the indispensable role of histone citrullination in the pathogenesis of RA. Additionally, neutrophils are involved in the development of systemic lupus erythematosus (SLE) by producing NETs, which contain dsDNA and nucleoprotein [27]. Interestingly, although NETs are associated with the development of both RA and SLE, SLE patients produced various components of NET proteins, including histones H1.0, H2B and H4 [28]. Furthermore, histone citrullination and NETs are found in the crypt abscess of the colon in patients with ulcerative colitis [29]. Additionally, PAD4 was reported to increase the histone citrullination of nucleosomes in normal white matter (NAWM) of multiple sclerosis (MS) patients and demyelinating animal models. PAD4-mediated histone citrullination could lead to irreversible changes in oligodendrocytes, resulting in the apoptosis of MS oligodendrocytes [30]. Furthermore, smoking has been shown to alter histone modification, DNA methylation patterns and miRNA expression, increasing the susceptibility to MS through epigenetic modification [31]. Many autoimmune diseases are associated with increased PAD and citrullination. However, researchers have found that citrullination also plays an important role in the development of tumors in recent years. In this study, the role of histone citrullination in tumors was elucidated. Additionally, considering the role of citrullination by PAD in physiological and pathological processes, PAD inhibitors have been studied and applied. Many PAD inhibitors are used to treat PAD disorders in the skin, joints, colon and immune system [32–35]. Compared with the parent F-amidine and Cl-amidine, the second generation of BB-Cl-amidine has better isozyme specificity, stability, and bioactivity in vivo.

In general, histone citrullination has received increasing attention because of its significance in both pathological and physiological conditions. Abnormal citrullination and histone citrullination regulation can provide new targets to treat various diseases.

## Peptidyl arginine deiminase family and citrullination

### PAD family

**Fig. 1:**
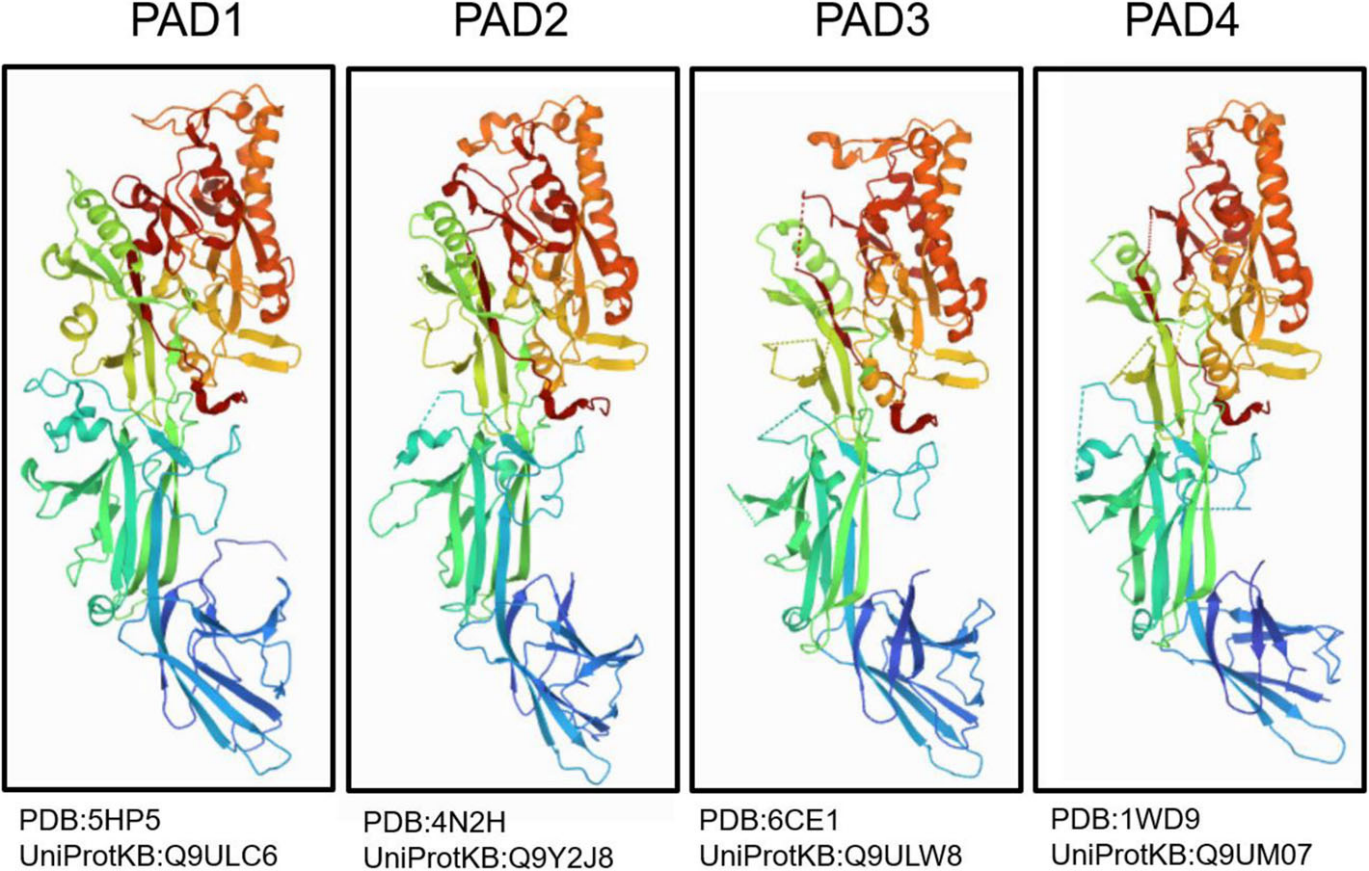
Structures of PAD1, PAD2, PAD3 and PAD4

PAD was first reported in 1977 as the citrullination enzyme during hair growth, and its substrate is the protein trichohyalin [36]. Five PAD enzymes have been identified in the human body: PAD1, PAD2, PAD3, PAD4 and PAD6 [37–39]. PAD enzymes have a specific tissue distribution and various catalytic substrates (Table 1) [59]. Both PAD1 and PAD3 are distributed in the hair follicles and epidermis [60]. Additionally, a high level of PAD1 was expressed in the uterus [39, 40]. PAD2 and PAD4 are widely distributed throughout tissues. For example, PAD2 is located in the spleen, central nervous system, skeletal muscles, and leukocytes [44, 61–63]. However, PAD4 is found in inflammatory cells (neutrophils and macrophages), breast cells, and tumor cells [35, 64, 65]. PAD6 is located in early embryos, eggs and ovaries [38, 65, 66]. Recently, the structure of PAD has been further studied. All the mammalian PAD isoenzymes have 70–95% homology in the amino acid sequence, with approximately 663 amino acids and a molecular weight of 74 kDa [50, 67–69]. The structures of PAD1, PAD2, PAD3 and PAD4 have been identified (Fig. 1). PAD2, PAD3 and PAD4 are homodimers, whereas PAD1 is a monomer in solution. Crystallization and preliminary X-ray crystallographic analysis of human PAD3 have been studied, and the structures of PAD family have been uncovered more comprehensively [70].

**Table 1:** Overview of PAD expression

Enzyme	Location	Substrates
PAD1	Epidermis, uterus [40, 41]	Keratin, filaggrin [20]
PAD2	Brain, uterus, spinal cord, salivary gland, macrophages, pituitary gland, sweat gland, spleen, pancreas, bone marrow, oligodendrocytes, yolk-sac (leucocytes) [41–43]	Myelin basic protein [44], vimentin [45], actin, histones [46]
PAD3	Hair follicles [41]	Filaggrin, trichohyalin [20], apoptosis-inducing factor, vimentin [47]
PAD4	Eosinophils, neutrophils, granulocytes [48, 49]	Histones [50, 51], Collagen, ING4 [52], p300 [53], nucleophosmin [54], p21 [55], Lamin C [56], RPS2 [57], DNMT3A
PAD6	Egg, ovary, early embryo [58]	None known

### Citrullination

**Fig. 2:**
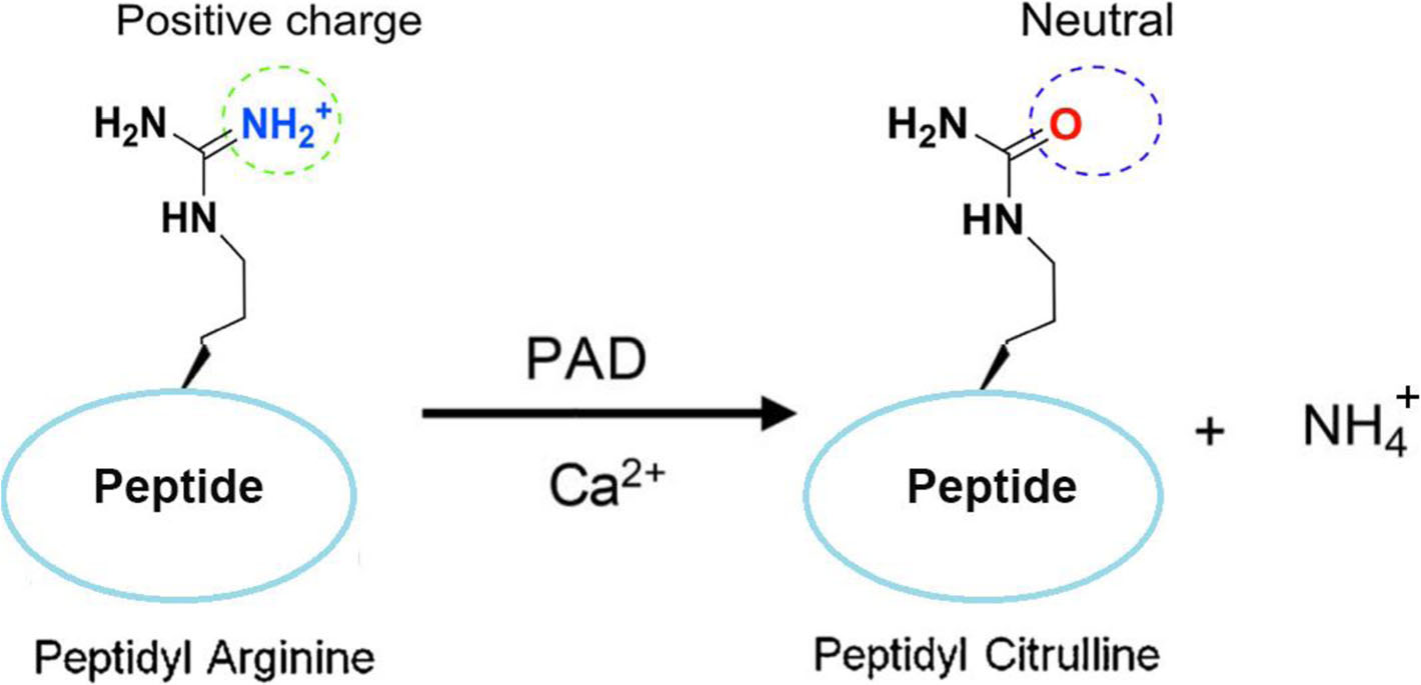
PAD-mediated citrullination

Citrullination is the Ca^2+^-driven enzymatic conversion of arginine residues to citrulline, catalyzed by the PAD family [17]. Citrullination or deimination indicates modification of the primary ketimine group (=NH) to a ketone group (=O), yielding ammonia as a side-product (Fig. 2) [50]. Thus, the strongly alkaline and positively charged arginine side chains are hydrolyzed to form a neutral urea. Charge transfer affects protein-protein interactions, hydrogen bond formation and protein structure, which may cause denaturation in some cases [13, 71]. Various proteins, such as cytoplasmic, nuclear, membrane and mitochondrial proteins, can be citrullinated [57]. Citrullinated proteins can be detected using antibody-based detection systems. The anti-citrulline modified (ACM) detection kit produced by Millipore is widely used to detect citrullinated proteins. The phenylglyoxal-based probe is used to detect citrullinated proteins, has a lower detection limit and can be easily used in a high-throughput assay format [72]. Additionally, mass spectrometry is the unique method that can identify the exact citrullinated site, particularly for high-abundance proteins.

### Citrullination in nonhistone proteins

Considering the diverse protein types, we demonstrated the functions and applications of nonhistone citrullination according to the sequence of the PAD family. PAD1 has been reported in keratin and filaggrin and plays a specific role in epidermis differentiation [73]. PAD2-mediated citrullination of MBP increases as inflammatory demyelination progresses in EAE and MS patients and is positively associated with diffuse inflammation in the brain [74]. The citrullination of vimentin mediated by PAD2 is significant in RA, with high sensitivity and specificity for the autoantibody reactivity against mutated and citrullinated vimentin (MCV). Furthermore, citrullinated vimentin was found in the brain tissues of patients with Alzheimer’s disease (AD) and sporadic Creutzfeldt-Jakob disease (sCJD) [34, 75]. PAD2-catalyzed actin and vimentin were found in DCs and DC-derived osteoclasts (OCs), which promote differentiation plasticity toward the OC lineage [76]. PAD3 can citrullinate filaggrin and trichohyalin, resulting in epidermal differentiation [77]. PAD3 was identified to balance the survival/death of human neural stem cells through the citrullination of apoptosis-inducing factor [47]. Regarding other PAD family members, PAD4 has more catalytic substrates. Collagen citrullination, mediated by PAD4, decreases the adhesion of synovial fibroblasts and mesenchymal stem cells, modifying the pathogenesis of RA [78]. PAD4 also deiminates nonhistone proteins such as p300, nucleophosmin (NPM1), ING4 and Lamin C, which are involved in cell apoptosis or DNA damage [79]. PAD4 can catalyze the ribosomal protein S2 (RPS2) region, which is the same site for protein arginine methyltransferase 3 (PRMT3). This finding is consistent with the role of citrullination in regulating RPS2 and ribosome assembly [57]. DNA methyltransferase DNMT3A can be citrullinated by PAD4, which regulates genomic CpG methylation [43].

In summary, nonhistone citrullination is more closely associated with autoimmune diseases such as RA. Nonhistone citrullination is involved in regulating biological function through histone modification. Histone citrullination contributes in various aspects to cancer development, which is discussed later.

## Histone citrullination

Considering the influence of PTM cross talk, we discussed the functions of histone citrullination in two parts, the physiological regulation of histone citrullination (Table 2) and cross talk between histones undergoing citrullination and other proteins undergoing PTM.

**Table 2:** Roles of histone citrullination in physiological regulation

Function	Enzyme	Citrullination site	Cell type	Details
Embryonic development	PAD1	H4R3, H3R2, 8, 17	Embryonic cells	Transactivation of the early embryo genome [80]
	Unknown	H3R2, 8, 17, H3R26, H4R3	Zygotes	Upregulation of gene expression in early embryos [81]
	PAD2	H3R2, 8, 17, H3R26	Ovine luminal epithelial	Gestation establishment [82]
Wound healing	PAD2	H4R3	Leukocyte in zebrafish	Novel signaling of regenerative growth [83]
Senescence	Unknown	H1.0	diploid fibroblasts	Regulation of aging associated with heterochromatinization [84]
Reproductive function	PAD2	H3R2, 8, 17	Mammary gland epithelial	Regulation of lactation gene expression [85]
	PAD2	H3R2, 8, 17	Gonadotrope cell	Regulation of gonadotropin gene expression [86]
Chromatin activity	PAD4	H1R54	Embryonic stem cell	Changes in histone-DNA interaction and regulation of chromatin accessibility [87]
	PAD4	H4R3	Thymus	Promotion of chromatin decondensation and DNA fragmentation [56]
	Unknown	H3R26	Embryonic stem cell	SMARCAD1 regulates pluripotency by interacting with CitH3R26 [88]
Pluripotency	PAD4	H1.2R53	Stem cell line	Regulation of pluripotency and chromatin decondensation [5]
Transcription	PAD4	H3R2,8,17,26 H4R3, H2A	HL-60	Antagonism against histone methylation [79]
NET formation	PAD4	H3	Neutrophil	Antibacterial natural immunity [89]
	PAD2	H3	Neutrophil	PAD2 inhibition reduces NETosis and inflammatory cytokine [90]
	PAD4	H3	Neutrophil	NET formation in the patient with COVID-19 [91]

### Role of histone citrullination in physiological regulation

Although PAD is considered a transcriptional regulatory protein that affects gene expression, the exact biological function of citrullination remains unclear [92]. Citrullinated histones account for approximately 10% of all histone molecules in HL-60 granulocytes, highlighting the significance of PTM in many nucleus-related processes [54]. Although few articles have described the proportion of citrullinated histones in other cells, histone citrullination exists in multiple cells from multiple systems, either health or disease, indicating the importance of histone citrullination regulation.

First, regarding embryonic development, PAD1 is mainly expressed in the epidermis and uterus. However, the role of PAD1 in preimplantation development has not been clarified. Zhang et al. significantly reduced the citrullination of H4R3 and H3R2/8/17 in embryonic cells using a specific inhibitor of PAD1. Consistent with this observation, early embryonic development was also arrested at the 4-cell stage. Additionally, PAD1 inhibition led to a sharp reduction in the overall transcriptional activity, which was associated with decreased phosphorylation of RNA polymerase II. These data revealed a new function of PAD1 in early embryonic development, facilitating transactivation of the early embryonic genome through histone citrullination [80]. Considering the observations of the effects of Cl-amidine and relationship between histone acetylation and transcriptional activation in embryonic development, another study suggested that histone citrullination promotes gene expression by creating a relaxed environment for chromatin histone acetylation [81]. PAD has been found in rodent uterine epithelial cells, which are associated with embryonic development. Examination of caruncle lysates from pregnant ewes and ovine luminal epithelial (OLE) cell lines showed that histone H3 arginine residues 2, 8, 17 and 26 were citrullinated. Next, pan-PAD inhibitors were used to treat OLE cells, resulting in a significant decrease in the expression of insulin-like growth factor binding protein 1 (IGFBP1) mRNA. Because of the effect of IGFBP1 on the migration and attachment of the trophectoderm to the endometrium, PAD-mediated citrullination was considered a crucial PTM for pregnancy [82].

In the model of tissue injury of zebrafish, PAD2-mediated H4Cit is necessary for efficient regeneration and was identified as a potential intermediary between early calcium signaling and subsequent wound healing [83].

Numerous similarities exist between senescence and differentiation. One key similarity is that chromatin remodeling events occur. The citrullinated linker H1.0 accumulates in aging cells and participates in heterochromatinization and aging [84].

To gain more insight into the potential reproductive role of PAD, PAD2 was expressed and localized in canine mammary epithelial cells. Additionally, the N-terminus of histone H3 was identified as the primary target for PAD activity in the breast epithelium, as indicated by site-specific antibodies against citrullinated histones. These results support the possibility that PAD2 regulates lactation-related gene expression through histone citrullination [85]. Furthermore, the GnRH agonist can catalyze histone H3 by stimulating PAD2, thereby epigenetically regulating the expression of gonadotropin genes such as LHβ and FSHβ [86].

PAD enzymes are extensively involved in regulating chromatin activity through histone citrullination. Recently, citrullination was reported to alter histone-DNA interactions. As a highly conserved globular region, the linker histone H1 is not a part of the core particle in embryonic stem (ES) cells and was found to be citrullinated at Arg54 [5]. However, this residue is required for histone interaction with nucleosomal DNA [93]. This observation at least partially explains why chromatin is more accessible in ES cells than in differentiated cells [87]. Histone modification is an essential mechanism for regulating chromatin dynamics [94]. PAD4-mediated citrullination can affect chromatin structure by promoting chromatin decondensation and DNA fragmentation [56, 95].

Histone citrullination regulates various cellular processes, particularly pluripotency. SMARCAD1 was found to preferentially bind CitH3R26 in preimplantation embryos, and this interaction inhibited the formation of heterochromatin, thereby regulating naïve pluripotency [88]. PAD4 can regulate the pluripotent transcription network by binding to the regulatory elements of key stem cell genes. Inhibition of PAD4 reduced the proportion of pluripotent cells in early mouse embryos and significantly decreased reprogramming efficiency [96]. Within the DNA-binding site of H1, citrullination of a single arginine residue led to total chromatin decondensation. In summary, these results reveal the roles of histone citrullination in pluripotency and provide new insights into the mechanism of regulating chromatin compaction [5].

Another function of citrullinated histones is altering the formation of NETs [26, 97]. As innate immune cells, neutrophils are the first responders to bacterial infections and contribute significantly to the defense against various pathogens, secretion of microbicidal agents and recruitment of other immune cells [97]. During the trapping of bacteria, neutrophils secrete cell-free DNA, histones and intracellular proteins into the extracellular space or circulatory system, forming so-called NETs. The PAD4-mediated citrullination of histones is involved in forming NETs. In contrast to PAD4^+/+^ neutrophils, PAD4^−/−^ neutrophils cannot form NETs after chemokine stimulation or incubation with bacteria and fail to kill bacteria by NETs, illustrating that PAD4 is critical for NET-mediated antimicrobial function [89]. However, recent studies have discovered that PAD2 inhibition reduces NET formation and inflammatory cytokine production in endotoxemia [90]. Additionally, the number of NETs remnants is increased in the sera of patients with COVID-19, and serum samples from COVID-19 patients clearly show that triggered healthy neutrophils undergo NETosis [91].

In summary, histone citrullination significantly contributes to embryonic development, reproductive function, chromatin expression, desorption, pluripotency and NET formation.

### Cross talk between citrullinated histones and histones with other modifications

During histone modification, citrullinated histones engage in cross talk with histones marked with other modifications. Wang et al. [22] and Cuthbert et al. [98] demonstrated that PAD4 is closely involved in the control of histone arginine methylation during transcription. Wang et al. found that calcium-activated PAD4 reduced the methylation of recombinant histone H3 and H4 in vitro. PAD4 worked by converting methyl arginine residues to citrulline and releasing methylamine. Wang et al. demonstrated this finding by isolating radiolabeled methylamine from PAD4-treated histone H4. The results showed that PAD4 is recruited to estrogen-responsive gene promoters and effectively inhibits estradiol-dependent transcription. Additionally, Cuthbert et al. enhanced our understanding of the regulation of arginine methylation by histone citrullination [98]. First, histone 3 undergoes citrullination only on residues 1–26, where histones most frequently undergo PTM. Second, Cuthbert et al. demonstrated that PAD4 recruitment to the pS2 promoter is associated with a transient increase in CitH3 following estrogen stimulation. Both research groups proposed a similar model in which PAD prevents histone arginine methylation either by directly citrullinating arginine or converting a monomethyl arginine to citrulline. In this scenario, monomethylated histones represent a “poised” state that makes histones resistant to citrullination. These histones are either demethylated to block the promoter or demethylated to activate the promoter. These findings are significant because they indicate that histone citrullination antagonizes arginine methylation.

Both Arg26 and Lys27 of histone H3 can be modified by various enzymes that have profound effects on gene expression [99]. Concerning regulating downstream genes, PAD2-mediated CitH3R26 has the opposite effect of PRC2-mediated H3K27 methylation. CitH3R26 activates gene expression, while H3K27 methylation inhibits gene expression. These modifications are driving factors for various cancers. After biochemical and cell-based analyses of these modifications, H3K27 methylation was found to slow H3R26 citrullination by 30-fold, while H3R26 citrullination slowed H3R26 methylation by 30,000-fold. Studies on this cross talk showed that the structure of the histone tail changed after citrullination, preventing the methylation of the PRC2 complex. Finally, researchers proposed a model in which H3K27 demethylases are recruited to chromatin to activate transcription after forming H3Cit26. In conclusion, the results support a cross-talk relationship between H3R26 undergoing citrullination and H3K27 undergoing methylation [99].

In addition to arginine methylation, histone citrullination cooperates with deacetylated histones to inhibit transcription. PAD4 was demonstrated to activate histone deacetylase 1 (HDAC1) to produce a repressive chromatin environment at the pS2 promoter [100]. Furthermore, PAD4 and HDAC2 interact with p53 through distinct domains and simultaneously bind to the p21 promoter to regulate gene expression. The PAD4 inhibitor (Cl-amidine) and HDAC inhibitor (suberoylanilide hydroxamic acid) have additive effects to increase the expression of p21, GADD45 and PUMA, which inhibit the growth of cancer cells dependent on P53 [92]. These studies indicate the dynamic cross talk between histones undergoing citrullination and deacetylation. In summary, the citrullination of histones can interfere with other PTMs and regulate gene expression.

## Functions of histone citrullination in tumors

**Fig. 3:**
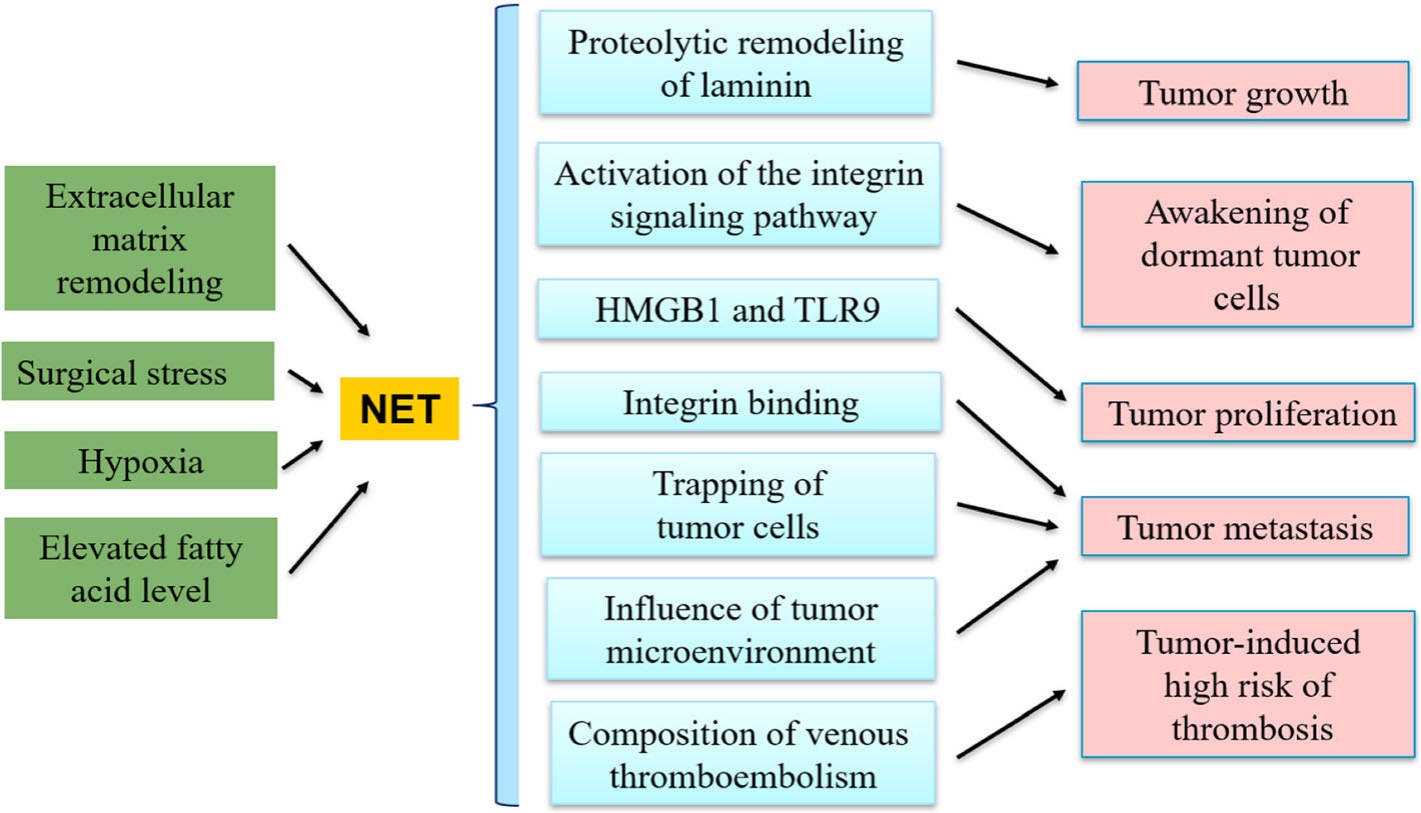
Mechanism by which NETs promote tumors

According to the classification system established by the World Health Organization (WHO), tumors can be mainly categorized into lymphatic hematopoietic tissue, endocrine, lung and mediastinum, digestive system, urinary and male reproductive, mammary gland, head and neck, central nervous system, skin, bone and soft tissue. In this review, the relationship between histone citrullination and tumors was elucidated based on the WHO tumor classification (Table 3). The specific role of histone citrullination-mediated NETs in tumors was discussed separately (Fig. 3). Additionally, the relationship between citrullination and the signaling pathway in tumor was described (Table 4).

**Table 3:** Histone citrullination in tumors

Classification	Disease	PAD-Citrullination	Mechanism
Lymphatic hematopoietic tissue	MM	PAD2- CitH3R6	Resistance to the chemotherapy drug bortezomib [101]
	Acute myeloid leukemia	PAD4-H3	Facilitation of the differentiation of HL-60 into granulocytes [102]
Endocrine system	Prolactinoma	PAD2, PAD4- CitH3R2, 8, 17	Inhibition of the expression of the miRNA targeted to the prolactin tumor oncogene [103]
Lung and mediastinum	Lung cancer	PAD4- CitH4R3	Inhibition of the transcriptional activity of p53 [56]
	Non-small cell lung cancer	PAD4- CitH4R3	Participation in the DNA damage response [79]
Digestive system	Gastric cancer	PAD4- CitH3R26	Interaction between H3R26Cit and H3K27me3 [79]
	Hepatocellular carcinoma	CitH3	Increase of Beclin1 mRNA during the development of hepatocellular carcinoma [104]
Urinary and male reproductive	Bladder cancer	Unknown	HSP90 inhibitor-mediated treatment [105]
	Prostate cancer	PAD2- CitH3R26	Upregulation of estrogen-suppressing genes [106]
Mammary gland	MCF-7	PAD4- CitH3R17	Reduction in β-estradiol-induced genes [22]
	MCF-7	PAD2-CitH3R2, 8, 17	Regulation of gene expression (PTN and MAGEA12) [107]
Bone and soft tissue	Osteosarcoma	PAD4- CitH3	Inhibition of the expression of the OKL38 gene [108]

**Table 4:** Citrullination and cell signaling in cancer

Biological process	PAD	Citrullinated Cite	Cell Signaling	Regulating process
EMT	PAD1	MEK1	ERk1/2 P38 MAPK	MMP2↑ [109]
	PAD4	GSK3β	TGF-β	Vimentin↑ E-cadherin↓ [110]
	PAD4	unknown	unknown	Elk1↓ [111]
Proliferation	PAD2	RNAP2 R1810	transcription	Altering gene expression [112]
	PAD4	Histone H3	HMGB1 TLR9	NET↑ [113]
	PAD2	β-catenin	Wnt/β-catenin	Wnt signaling↓ [114]
Metastasis	PAD4	Histone H3	Integrinα-3β-1	NET↑ NE↑ MMP9↑ [115]
	PAD4	Histone H3	HMGB1 TLR9	NET↑ [113]
	PAD4	Histone H3	TLR4/9-COX2	NET↑ [116]
Apoptosis	PAD4	ING4	P53	P21↓ [52]
	PAD4	OKL38	P53	Histone methylation↓ [108]
DNA damage	PAD4	Histone H3	P53	Regulating gene expression [92]
	PAD4	NPM1	P53	growth-inhibitory activity↑ [117]
	PAD4	Histone H4R3	P53	chromatin decondensation and DNA cleavage↑ [56]
Autophagy	unknown	MHC-II	Antigen recognition	Antitumor immunity↑ [118]
	unknown	Histone H3	HMGB1	NET↑ [119]

### Histone citrullination in tumors

In lymphatic hematopoietic tissue, mutant malignant cells cannot differentiate into healthy granulocytes; instead, they undergo uncontrolled self-replication characteristics of acute promyelocytic leukemia. A recent study showed that PAD4 expression is elevated during the differentiation of HL-60 leukemia cells induced by all-trans retinoic acid (ATRA), and the differentiation of HL-60 cells depended on demethylation of the PAD4 promoter [102]. PAD4 stimulated the expression of the hematopoietic transcription factors sox4 and pu.1 through histone citrullination, facilitating the differentiation of HL-60 cells into granulocytes [102]. This work highlighted the significance of PAD in cell differentiation, a finding supported by another extensive embryonic study in which PAD4 and histone citrullination were identified as major regulators of pluripotency [5]. Multiple myeloma (MM) is a malignancy of plasma cells located in the bone marrow. The MM environment promotes critical interactions between cancer cells and stromal cells, facilitating the survival and proliferation of tumors. Increasing evidence has shown that bone marrow mesenchymal stem cells (BMMSCs) are stably altered in MM and monoclonal gammopathy of undetermined significance (MGUS). PAD2 transcripts were among the most upregulated transcripts in BMMSCs. Moreover, the activity of PAD2 directly induced the upregulation of IL-6 through CitH3R26. Subsequently, IL-6 enabled malignant plasma cells to acquire resistance to bortezomib. This is a newly discovered mechanism showing that the dysfunction that causes MGUS and MM in patients directly leads to promalignant signaling through CitH3R26.

In the endocrine system, the expression and function of PAD enzymes in pituitary adenoma remain unclear. PAD2, PAD4 and citrullinated histones are highly expressed in prolactinomas and somatoprolactinomas. RNA sequencing and ChIP studies showed that histone citrullination inhibited the expression of miRNAs let-7c-2, 23b, and 29c. These miRNAs directly target the oncogenes of HMGA, insulin-like growth factor-1 (IGF-1) and N-MYC and are highly correlated with the pathogenesis of human prolactinoma/somatoprolactinoma. Histone citrullination regulates miRNA expression, revealing the etiology of prolactinoma and somatoprolactinoma [103].

In the lung and mediastinum, PAD4 interacts with p53 to induce CitH4R3 after chemotherapy treatment [56]. The colocalization of citrullinated regions and decondensed chromatin in apoptotic cells indicated that the PAD4-p53 complex is involved in apoptosis, while PAD4-knockout mice showed apoptotic resistance. Additionally, lung cancer patients had smaller tumors than those without CitH4R3. Many studies have focused on the relationship between PAD4 and p53. PAD4 binds to the C-terminus of p53 and participates in inhibiting p53 transcription, indicating that PAD4 is a coinhibitory factor of p53. Histone arginine methylation contributes significantly to p53 target gene activation. P53 recruits PAD4 to the promoter of the p53 genes and reverse methylation, inhibiting the expression of p21/CIP1/WAF1 and OKL38 [108]. H4R3 depends on p53/PAD4 activated in the DNA damage response and is negatively correlated with p53 protein expression and tumor size in non-small cell lung cancer [79]. These results indicate that PAD4 is involved in tumor development by regulating the transcriptional activity of p53.

In the digestive system, IPO-38 is a potential biomarker for the early diagnosis of gastric cancer. The anti-IPO-38 antibody, which is biomarker, is thought to be histone modified. Next, the researchers identified the sites of histone modifications and labeled them with an anti-IPO-38 monoclonal antibody. In the protein array analysis, the citrullination-modified signal of CitH3R26 was the strongest. Although PAD2 and PAD4 catalyze citrullination, only PAD4 expression is associated with CitH3R26 in gastric cancer cell lines. The interaction between CitH3R26 and H3K27me3 was detected by immunoprecipitation and western blotting. These new findings suggest that the citrullination of histones plays a crucial role in gastric cancer and may help optimize and develop a sensitive diagnostic reagent [120]. Additionally, PAD4 contributes to the metastasis of gastric tumors by regulating the expression of CXCR2, KRT14 and TNF-β, which can stimulate angiogenesis, cell proliferation and migration, and tumor immune microenvironment establishment [121]. A PAD4 inhibitor was demonstrated to induce the differentiation of HT29 colon cancer cells [122]. Researchers detected CitH3 in hepatitis B virus-associated hepatocellular carcinoma (HCC) tissues and assessed its association with Beclin1 (a key autophagy regulator) mRNA. The average levels of H3Cit and Beclin1 mRNA in HCC were higher than those in nontumor tissues and were significantly correlated with vascular invasion and serum AFP levels. These results suggest that CitH3 is related to the increase in Beclin1 expression during the development of HBV-associated HCC [104].

Regarding the male urinary tract and reproduction, citrullination is involved in the androgen signaling pathway in prostate cancer [106]. Castration after long-term androgen deprivation therapy remains a major obstacle to treating prostate cancer. The results confirmed that PAD2 is an androgen suppressor gene that is increased in prostate cancer. PAD2 expression is required for the survival and cell cycle development of prostate cancer cells, whose proliferation is promoted. Cytoplasmic PAD2 protects androgen receptor (AR) against proteasome-mediated degradation and promotes AR binding to target genes through CitH3R26. PAD2 controls AR, depending on its enzyme activity and nuclear localization, which is associated with increased CitH3 expression. Notably, the coadministration of PAD inhibitors and AR signal transduction inhibitors inhibited cell proliferation and tumor growth in prostate cancer. Overall, PAD2-mediated histone citrullination is a potential therapeutic target for prostate cancer [106].

In the mammary gland, MCF-7 breast cancer cells showed a dramatic reduction in β-estradiol-induced genes after CitH3 was induced by PAD4, altering the cell phenotype [22]. Subsequently, Kuhenrod’s team found that the loss of PAD2 in MCF-7 breast cancer cells led to a disorder in a unique gene subset. PAD2 directly bound to these unique gene promoters and upregulated or downregulated their expression through the citrullination of H3R2, H3R8, and H3R17 [107]. Additionally, PAD4 specifically citrullinated residues H3R2, H3R8, H3R17, and H3R26 in HEK293 and MCF-7 breast cancer cells [98].

In bone and soft tissue, the expression of the p53 target gene OKL38 is inhibited by the recruitment of PAD4 to the OKL38 promoter. PAD4 subsequently removes the histone arginine methylation mark, directly regulating apoptosis [123]. The multiple interactions between PAD4 and p53 suggest the importance of PAD4-induced citrullination in apoptosis. In particular, Cl-amidine increased the expression of the OKL38 gene in osteosarcoma cells, leading to cell apoptosis, mitochondrial structural changes and the release of cytochrome c [108]. Additionally, histone deacetylase 2 (HDAC2) interacts with PAD4. Both HDAC2 and PAD4 bind to p53 and the p21 promoter in response to DNA damage, resulting in the regulation of gene expression [92]. Furthermore, inhibition of PAD4 and HDAC2 affected the histone modification of p53 target gene promoters and inhibited osteosarcoma growth in a p53-dependent manner [92]. However, various views persist regarding how PAD inhibitors induce apoptosis in U2OS osteosarcoma cells [124, 125]. These perspectives may be based on the cell type-specific role of PAD4 in apoptosis [126].

In summary, various tumors are associated with the hyperexpression of PAD and increased citrullination. The challenge is identifying specific citrullinated proteins involved in various autoimmune diseases. Additionally, protein identification is the basis for further study of the mechanism of citrullination mechanisms. As mentioned above, histone citrullination affects tumorigenesis in various ways, such as regulating gene transcription, cell differentiation and apoptosis. The discovery of histone citrullination, a reaction in which histones are substrates for PAD, may be a breakthrough in tumor research.

### NETs and tumors

The first evidence for the release of chromatin from neutrophils into the extracellular space was provided by Arturo Zychlinsky’s group in 2004 [97] who found that activated neutrophils produce extracellular DNA fibers and partial nuclear components to capture and kill bacteria, defining a previously unknown form of an innate antimicrobial response. Further studies showed that NETosis is a novel cell death process that leads to the decondensation of chromatin. This process includes disintegration of the nucleus and granular membrane, mixing of the cellular components, cytoplasmic membrane lysis and NET release [127]. The release of chromatin by neutrophils was initially shown to depend on the production of reactive oxygen species (ROS) [127]. These ROS are generated through the activation of NADPH oxidase, RAF-MEK-ERK and p38 MAPK pathways. Other factors activate NET formation in an NADPH oxidase-independent manner. However, other studies showed that neutrophil elastase and myeloperoxidase are involved in this process [128]. Neutrophil elastase can be transferred to the nucleus and facilitates chromatin nucleation by partially degrading histones.

Although the molecular mechanism for NET formation is unclear, histone citrullination strongly affects NET formation. The PAD4 enzyme is highly expressed in many cancers and neutrophils and is essential for NET formation [89]. PAD4 plays a role in chromatin decondensation to form NETs through the citrullination of histone H3 [89]. Additionally, PAD4 overexpression in the osteosarcoma cell line is sufficient to induce chromatin decondensation and release, demonstrating its function in this artificial environment [95]. It remains to be clarified how to regulate the expression and activity of PAD4 in neutrophils and why NET causes nuclear release or cell lysis. Many studies have confirmed that only PAD4 can deiminate histones inside the nucleus of neutrophils because it bears the nuclear localization signal. However, Wu et al. found that PAD2 inhibition reduces NET formation, decreases inflammatory cytokine production and protects against endotoxin-induced lethality [90]. In addition to CitH3, the citrullination of H1.0, H1.4, H1.5 and H2B was also detected in RA NET [28]. Because of its quantifiable response to all kinds of stimuli of NETs, CitH4 was identified as an index of early NETs [129]. In conclusion, both core histones (H2, H3 and H4) and linker histone H1 present citrullination in NETs. Although the citrullination of histones is associated with chromatin decondensation, which is associated with NET formation, the exact mechanism remains to be studied [130].

NETs have been detected in several types of human cancer and are suspected to promote cancer development [113, 131]. Chronic inflammation is thought to activate dormant malignant cells, but the mechanism remains unclear [132]. Recently, in an experimental lung cancer tumor model, inflammation-induced NETs activated dormant cancer cells by activating the integrin signaling pathway in tumor cells [133]. In particular, NET-derived DNA acted as a proteolysis scaffold by releasing elastase and MMP9, promoting cancer cell proliferation through bioactive epitopes in cleaved laminin. These findings indicate that chronic inflammation may cause the recurrence of cancer after a long period of dormancy. In another study, the level of NETs in a lung cancer-transplant tumor model increased by 35% compared with that in PAD4-knockout mice (no NETs). B16-transplanted tumors grew more slowly than Lewis-transplanted tumors because fewer neutrophils were activated [134]. However, these neutrophils were initiated to undergo NET formation following the administration of G-CSF, which promoted NET production and increased the growth of B16 tumors [134]. However, the study failed to provide a mechanistic explanation for why cancers require NETs for growth. Further studies showed that NETs activated the Toll-like receptor 9 (TLR9) signaling pathway in cancer cells by releasing highly mobile group box 1 (HMGB1) proteins [135].

In addition to promoting the proliferation and growth of tumor cells, NETs can also serve as adhesive substrates to transport cancer cells via integrin binding [136]. In vitro, cultured tumor cells show enhanced adhesion to NETs and are destroyed by anti-integrin antibody preincubation [136]. Furthermore, in a sepsis mouse model, microvascular NET deposition trapped circulating lung cancer cells, significantly promoting metastasis [137]. This process was attenuated by the systemic use of NET inhibitors or neutrophil elastase inhibitors. After the injection of tumor cells, NET-mediated capture of cancer cells in the hepatic sinus was associated with an increase in the formation of hepatic micrometastases and an overall metastatic disease burden. Similarly, this study demonstrated that human and mouse tumor cells could be captured by NETs in vitro [14].

Dead neutrophils and reticular structures are widespread in the hemorrhagic tumors of Lewis lung cancer (LLC) [138]. These apparent necrotic areas lose their typical nuclear morphology and show neutrophil abundance and extracellular chromatin with citrullinated histones. A similar pattern was observed in two sections of tumor tissue obtained from patients with Ewing’s sarcoma [131]. Therefore, NETs are found at the site of tumor neutrophil aggregation, possibly affecting the tumor microenvironment.

In humans, neutrophil accumulation is mainly observed in renal cell carcinoma, melanoma, HCC, glioblastoma, colorectal cancer, gastric cancer, esophageal cancer, lung cancer, ovarian cancer and head and neck cancer, most of which are associated with a high risk of venous thromboembolism. The leukemoid reaction induces increased neutrophils in a mouse model of breast cancer, finally causing spontaneous thrombosis [139]. The formation of pulmonary thrombi was observed when the neutrophil and plasma DNA levels were dramatically increased in the late stage of thrombosis in the murine model. Surprisingly, analysis of the neutrophils in the blood of tumor-bearing mice revealed that an increasing number of highly citrullinated neutrophils appeared and formed NETs as the tumor progressed. In late thrombosis, many highly citrullinated neutrophils and abundant CitH3 were detected, indicating the spontaneous formation of NETs [140]. The thrombus state seemed to be associated with DNA scaffolds when NETs were generated [140]. Neutrophils and a NET biomarker (CitH3) can be used as diagnostic tools to evaluate thrombophilia. Further studies found that CitH3 was detected in the plasma of cancer patients with acute thrombotic microangiopathy (TMA). Furthermore, cell-free CitH3 was observed in the plasma of cancer patients with thrombotic complications. This finding highlighted the potential use of CitH3 as a biomarker for cancer-related thrombosis.

An explanation of the conditions that enable cancer NET formation would be interesting. Tohmeand et al. demonstrated that hemolytic neutrophils cultured in a medium conditioned with colon cancer cells produced NETs under hypoxic conditions characteristic of surgical stress [113]. Under surgical stress, tumor cells and colonies easily accumulate in the liver, while the administration of NET inhibitors significantly reduce the occurrence and growth of metastatic tumors. Genetic and pharmacological targeting of PAD4 showed similar results in liver metastases. These results suggest that anoxia after surgical stress may be necessary for NET generation in the tumor microenvironment. Consistent with these findings, hypoxia induced the production of citrullinated proteins in malignant tumor antiglioma cells [135]. In addition to hypoxia, elevated free fatty acids stimulate NET formation and promote the growth of HCC in mice [141].

In summary, histone citrullination-mediated NETs are linked to tumor growth and metastasis through ECM remodeling, surgical stress, hypoxia, fatty acid levels, and/or cell metastasis via physical binding. Consistent with this finding, tumor-infiltrating NETs predict a poor postoperative survival of tumor patients [142]. However, the role of NETs in tumor development requires further studies.

### Citrullination and cell signaling during EMT, proliferation, metastasis, apoptosis, DNA damage and autophagy

The epithelial-to-mesenchymal transition (EMT) induces tumor metastasis and drug resistance by decreasing epithelial polarity and adhesive properties [143]. In human triple-negative breast cancer, PAD1 induces EMT by inhibiting ERK1/2 and P38 MAPK signaling through the citrullination of MEK1 [109]. PAD4 catalyzes the citrullination of transcription factor glycogen synthase kinase 3 beta (GSK3β), which induces EMT by decreasing E-cadherin and increasing vimentin expression [110]. However, PAD4 represses EMT by inhibiting the expression level of Elk1 in lung cancer cell lines [111].

RNA polymerase II (RNAP2) regulates gene expression by transcription elongation. PAD2 citrullinates RNAP2 in breast cancer cells, inducing the transcription of thousands of genes and activating cell proliferation [112]. NETs trigger high mobility group box 1 (HMGB1) release and activate the TLR9 pathway to facilitate tumor cell adhesion, proliferation, migration and invasion in colorectal cancer [113]. Additionally, citrullination of β-catenin mediated by PAD2 decreases the proliferation of colon cancer by repressing the Wnt-pathway [114].

The levels of NET-associated proteases, including neutrophil elastase (NE) and matrix metalloproteinase 9 (MMP9), are increased in cancer. They proteolytically remodel laminin to activate the signaling of integrin α-3β-1, promoting cancer cell proliferation and metastasis [115]. NETs catalyzed by PAD induce tumor inflammation by activating the TLR4/9-COX2 axis, thereby promoting liver cancer metastasis [116].

PAD4 and p53 have been extensively studied, revealing the interactions between PAD-induced citrullination and apoptosis. The interaction of PAD4 with p53 was colocalized with decondensed soluble chromatin in apoptotic cells, suggesting the involvement of the PAD4-pp53 complex in apoptosis [56]. The citrullination of ING4 at the nuclear localization sequence region inhibited p53-to-ING4 binding, decreased p53 acetylation, and subsequently repressed p21 expression [52]. PAD4 was recruited to the promoter of OKL38 by p53, which repressed the expression of OKL38. Inhibition of the p53 target gene OKL38 subsequently removed the histone arginine methylation mark, directly modulating apoptosis [108].

HDAC2 is a PAD4-interacting protein; both HDAC2 and PAD4 can bind to the p53 and p21 promoters, contributing to the regulation of gene expression in response to DNA damage [92]. Furthermore, the citrullination of nucleophosmin (NPM1) by PAD4 results in its translocation from the nucleoli to the nucleoplasm, increasing p53-mediated growth-inhibitory activity [117].

Stressful conditions in the tumor microenvironment induce autophagy in cancer cells. Autophagy plays an important role in presenting citrullinated peptides on MHC class II molecules to CD4^+^ helper T cells, suggesting that concomitant MHC class II expression and inflammation-induced IFN-γ promote immune activation [118]. Furthermore, receptor for advanced glycation end products (RAGE) promotes neutrophil autophagy, thereby enhancing NET formation in pancreatic cancer [119].

In conclusion, PAD-mediated citrullination plays an essential role in tumor development. The citrullination reaction can interfere with tumor EMT, metastasis, cell proliferation, autophagy and DNA damage processes by regulating various cell signaling pathways. Except for nonhistone proteins, histone citrullination participates in almost all biological processes, providing new methods to diagnose and treat tumors.

## Application of histone citrullination as a biomarker and therapeutic target

Regarding tumor immunity, new epitopes induced by PTMs may provide new targets for tumor-specific immunotherapy. Nutrient deficiency, hypoxia, redox stress, DNA damage and other tumor microenvironmental conditions can increase the activity of PAD and expression of citrullinated protein. Recently, the average CitH3 concentration in late malignant tumor patient serum increased three-fold compared with that in healthy people, indicating that citrullinated proteins may be cancer biomarkers [144]. In one group of cancer patients, the mean serum level of CitH3 in invasive tumors was higher than that in localized tumors, consistent with previous reports of PAD-mediated citrullination and metastasis [137]. Notably, the level of CitH3 in the plasma of cancer patients was associated with higher levels of cell-free DNA and neutrophil activation. Remarkably, researchers found that a high plasma level of CitH3 (> 29.8 ng/mL, more than 75%) is strongly associated with the risk of short-term death [144]. The increased expression of CitH3 is considered a novel prognostic blood marker in patients with advanced cancer [97]. The proportion of CitH3-positive neutrophils is increased in patients with more severe disease. The level of CitH3 in serum is closely related to neutrophil activation markers such as elastase, myeloperoxidase, IL-6 and IL-8. Therefore, CitH3 is considered a useful biomarker for evaluating the inflammatory response and prognosis of patients with advanced cancer.

The role of histone citrullination in cancer therapy has also been reported. Venous thromboembolism (VTE) often occurs during cancer treatment and can be life-threatening. Studies have shown that CitH3 is independently associated with VTE in cancer patients and plays an important role in predicting VTE occurrence in cancer treatment [145]. Additionally, inhibition of PAD2-mediated H3Cit26 decreases the expression of IL-6 in BMMSCs, mediating malignant plasma cell resistance to chemotherapeutic agents [101]. Song et al. found that an effective inhibitor of PAD can enhance antitumor activity by inhibiting CitH3 in an HCT-116 xenograft mouse model. Moreover, PAD2-H3Cit26 is considered a novel therapeutic target in castration-resistant prostate cancer [106]. Furthermore, cross talk between histones undergoing deacetylation and those undergoing citrullination is associated with cancer cell growth, indicating a combination of PAD and histone deacetylase inhibitors as a strategy for cancer treatment [92]. In MCF-7 cells, Cl-amidine regulates the expression of the tumor suppressor protein OKL38 by decreasing histone citrullination at the OKL38 promoter [108]. Finally, ubiquitous compounds used in herbal medicine inhibit hematogenous metastasis of certain tumors by targeting CitH3 and NETs [146].

Although it is difficult to determine the degree of citrullination in tissues and body fluids accurately [147], measuring the relative concentration of PAD enzymes appears to be a reliable method for estimating the citrullination level. PAD4 is significantly expressed in various malignant tissues but at a low level in both normal tissues and benign tumors [148]. PAD4 is elevated in various solid tumors and overexpressed in the peripheral blood of lung cancer patients [64, 149]. Approximately 40% of malignant lymphoma cells also express PAD4, indicating that PAD4 expression is associated with cancer development across all embryological lineages. The level of PAD4 is significantly higher in metastatic foci than in the corresponding primary tumor [150], indicating that citrullination may participate in the progression of benign tumors to aggressive malignancies. PAD4 is involved in tumor formation through the citrullination of histones, cytokeratin, antithrombin and fibronectin [151]. Although tumor-associated PAD2 expression was increased in patients with castration-resistant prostate cancer, the downregulation of intertumoral PAD2 expression was identified in patients with colorectal cancer [106, 152]. Because PAD2 and PAD4 are expressed at a higher level than other isozymes in humans, other members of the PAD family in cancer tissue have not been widely investigated. Therefore, these data suggest that PAD-mediated citrullination may be a biomarker or recognition target for cancer therapy.

Several research groups have used bioinformatics to predict citrullinated sites. Ju and Wang provided a user-friendly web server for CKSAAP-CitrSite [153]. Zhang et al. analyzed the citrullinated sites and constructed a classifier using a random forest algorithm [154]. Bioinformatics is believed to provide some useful insights into the study of citrullination. In conclusion, increasing attention has been directed to the application of histone citrullination as a diagnostic marker and therapeutic target (Table 5).

**Table 5:** Application of histone citrullination in cancer

Application value	PAD	Citrullinated cites	Evidence	Tumor types
Biomarker	PAD4	Histone H3	CitH3 was higher in patients with advanced cancer and predicted poor clinical outcomes and high short-term mortality.	Adenocarcinoma [144]
Biomarker	PAD4	Histone H3	CitH3 could predict the occurrence of venous thromboembolism in cancer treatment.	Unspecified [145, 155]
Therapeutic targets	Unknown	Histone H2AX	Histone citrullination mediates the formation of NETs, which contribute to tumor metastasis in the context of systemic infection.	Lung cancer [137]
Therapeutic targets	PAD2	Histone H3R26	Citrullination of H3R26 leads to pro-malignancy signaling in multiple myeloma.	Multiple myeloma [101]
Therapeutic targets	PAD2	Histone H3R26	PAD2-H3Cit26 was a key mediator for androgen receptor in prostate cancer progression.	Prostate cancer [106]
Therapeutic targets	PAD4	Histone H3	PAD4 and HDAC2 inhibitors is a potential strategy for treating cancer.	Osteosarcoma [92]
Therapeutic targets	PAD4	Histone H3	Histone citrullination can regulate the expression of tumor suppressor gene OKL38.	Osteosarcoma [108]
Therapeutic targets	PAD4	Histone H3	Herbs can effectively inhibit hematogenous metastasis of tumors by targeting NETs (citrullination-mediated).	Gastric carcinoma [146]
Therapeutic targets	PAD4	Histone H3	Prevention of NETs in cancer can inhibit tumor-induced thrombosis and organ failure as well as inhibit metastasis.	Insulinoma and breast cancer [156]

## Application and development of PAD inhibitors

Given the important role of PAD-mediated citrullination in tumors, the application of PAD inhibitors has been investigated. PAD inhibitors such as Cl-amidine and F-amidine can trigger the differentiation and apoptosis of various cancer cells (including HL60, HT29, TK6, and U2OS cells). Additionally, PAD is overexpressed in cancer, indicating the significance of PAD inhibitors in treating tumors. PAD4 is the only member of the PAD family that contains a nuclear localization signal, and it can citrullinate various substrates, including histones. PAD4 inhibitors have been used with cancer patients to prevent tumor spread and cancer-related thrombosis [64]. Thus, PAD inhibitors can inhibit the proliferation of cancer cells and reduce tumor growth [92].

NET formation is a marker for several diseases, such as lupus, ulcerative colitis and RA [157]. PAD inhibitors may be used as a treatment for disorders associated with abnormal NET formation. The discovery of NETs in the tumor environment also provides new clues for the development of diagnostic tools. Recently, Wang and his colleagues designed an effective PAD4 inhibitor with a strong inhibitory effect on tumor growth. In addition to their direct effects on cancer cells, these PAD4 inhibitors prevented PAD4-mediated NETosis, which inhibited tumor growth and cancer-related thrombosis.

Although several reversible PAD inhibitors (paclitaxel, minocycline and streptomycin) have been identified, these compounds are relatively weak PAD inhibitors [158]. The low efficacy of these reversible inhibitors may be related to the small active site cavity, which may contain only side chains of arginine residues during the catalytic process. Given the lack of efficacy of these reversible inhibitors, Cl-amidine analogs have attracted attention for their improved efficacy, selectivity and bioavailability [159]. As second-generation PAD inhibitors, O-F-amidine and O-Cl-amidine have improved efficacy and selectivity. For example, O-F-amidine had a 65-fold stronger effect than F-amidine. Additionally, compared with F-amidine, O-F-amidine shows greater preferential PAD1 suppression. Cl-amidine is a pan-PAD inhibitor that inhibits several members of the PAD family. However, its high IC50 limits its preclinical exploration in cancer research and treatment [160]. Recently, Wang et al. identified YW3–56, which activates a group of p53 target genes and inhibits the mTORC1 signaling pathway. YW3–56 was demonstrated to obstruct autophagy and inhibit cancer cell growth. The properties of Cl-amidine are still being studied, and several new PAD small-molecule inhibitors have been developed by pharmacologists [161].

Taken together, these data demonstrate the feasibility of using PAD inhibitors to treat tumors. PAD inhibitors significantly reduce the proliferation of cancer cells without affecting the viability of normal cells [122], representing a potential pathway for targeted therapy. However, new inhibitors are needed to overcome some of the disadvantages of current PAD inhibitors, such as poor selectivity, low bioavailability and relatively mixed reactivity.

## Conclusion

Epigenetic modification supplements classical genetics, and modifications involving PAD-mediated histone citrullination play integral roles in physiology and tumors. In this review, we discussed the classification and characterization of the PAD family and the physiological regulation of chromatin structures and gene transcriptional activity by PAD-mediated histone citrullination. Additionally, histone citrullination in tumors was discussed based on WHO classification. Regarding tumors, NETs mediated by histone citrullination contributed to antibacterial innate immunity and tumor development. Finally, the application of histone citrullination and PAD inhibitors was discussed. Therefore, PAD-mediated histone citrullination is likely a promising tumor marker and therapeutic target in the future.

## Data Availability

The material supporting the conclusion of this review has been included within the article.

## Abbreviations

ACPA: Anti-citrullinated protein antibody
ACM: Anti-citrulline modified
AD: Alzheimer’s disease
ATRA: All-trans retinoic acid
AR: Androgen receptor
BMMSCs: Bone marrow mesenchymal stem cells
ES: Embryonic stem
EMT: Epithelial-to-mesenchymal transition
GSK3β: Glycogen synthase kinase 3 beta
HDAC1: Histone deacetylase 1
HCC: Hepatocellular carcinoma
HDAC-2: Histone deacetylase 2
HMGB1: High mobility group box 1
IGFBP1: Insulin-like growth factor binding protein 1
IGF-1: Insulin-like growth factor-1
LLC: Lewis lung cancer
MS: Multiple sclerosis
MBP: Myelin basic protein
MCV: Mutated and citrullinated vimentin
MM: Multiple myeloma
MGUS: Monoclonal gammopathy of undetermined significance
NET: Neutrophil extracellular trap
NAWM: Nucleosome in normal white matter
NPM1: Nucleophosmin
NE: Neutrophil elastase
OCs: Osteoclasts
OLE: Ovine luminal epithelial
PAD: Peptidyl arginine deiminase
PTM: Posttranslational modification
PRMT3: Protein arginine methyltransferase 3
RA: Rheumatoid arthritis
RPS2: Ribosomal protein S2
ROS: Reactive oxygen species
RAGE: Receptor for advanced glycation end products
SLE: Systemic lupus erythematosus
sCJD: Sporadic Creutzfeldt-Jakob disease
TLR-9: Toll-like receptor 9
TMA: Thrombotic microangiopathy
VTE: Venous thromboembolism

## References

[1] Kornberg RD (1974). Chromatin structure: a repeating unit of histones and DNA. Science.

[2] Kornberg RD, Klug A (1981). The nucleosome. Sci Am.

[3] Kornberg RD, Lorch Y (1999). Twenty-five years of the nucleosome, fundamental particle of the eukaryote chromosome. Cell.

[4] Cutter AR, Hayes JJ (2015). A brief review of nucleosome structure. FEBS Lett.

[5] Christophorou MA, Castelo-Branco G, Halley-Stott RP, Oliveira CS, Loos R, Radzisheuskaya A, et al. Citrullination regulates pluripotency and histone H1 binding to chromatin. Nature. 2014;507(7490):104–8. 10.1038/nature12942 PMC4843970 24463520

[6] Pérez-Montero S (2013). The embryonic linker histone H1 variant of Drosophila, dBigH1, regulates zygotic genome activation. Dev Cell.

[7] van Attikum H, Fritsch O, Gasser SM. Distinct roles for SWR1 and INO80 chromatin remodeling complexes at chromosomal double-strand breaks. EMBO J. 2007;26(18):4113–25. 10.1038/sj.emboj.7601835 PMC2230671 17762868

[8] Soboleva TA (2017). A new link between transcriptional initiation and pre-mRNA splicing: The RNA binding histone variant H2A.B. PLoS Genet.

[9] Yoda K (2000). Human centromere protein a (CENP-A) can replace histone H3 in nucleosome reconstitution in vitro. Proc Natl Acad Sci U S A.

[10] Chen P, Zhao J, Wang Y, Wang M, Long H, Liang D, et al. H3.3 actively marks enhancers and primes gene transcription via opening higher-ordered chromatin. Genes Dev. 2013;27(19):2109–24. 10.1101/gad.222174.113 PMC3850095 24065740

[11] Kinner A (2008). Gamma-H2AX in recognition and signaling of DNA double-strand breaks in the context of chromatin. Nucleic Acids Res.

[12] van Venrooij WJ, Pruijn GJ (2000). Citrullination: a small change for a protein with great consequences for rheumatoid arthritis. Arthritis Res.

[13] Tarcsa E (1996). Protein unfolding by peptidylarginine deiminase. Substrate specificity and structural relationships of the natural substrates trichohyalin and filaggrin. J Biol Chem.

[14] Audia JE, Campbell RM. Histone modifications and Cancer. Cold Spring Harb Perspect Biol. 2016;8(4):a019521. 10.1101/cshperspect.a019521 PMC4817802 27037415

[15] Bicker KL, Thompson PR. The protein arginine deiminases: structure, function, inhibition, and disease. Biopolymers. 2013;99(2):155–63.

[16] Huang H, Lin S, Garcia BA, Zhao Y. Quantitative proteomic analysis of histone modifications. Chem Rev. 2015;115(6):2376–418. 10.1021/cr500491u PMC4502928 25688442

[17] Rogers GE, Simmonds DH (1958). Content of citrulline and other amino-acids in a protein of hair follicles. Nature.

[18] Fujisaki M, Sugawara K (1981). Properties of peptidylarginine deiminase from the epidermis of newborn rats. J Biochem.

[19] Inagaki M, Takahara H, Nishi Y, Sugawara K, Sato C. Ca2+−dependent deimination-induced disassembly of intermediate filaments involves specific modification of the amino-terminal head domain. J Biol Chem. 1989;264(30):18119–27. 2808368

[20] Senshu T (1995). Detection of deiminated proteins in rat skin: probing with a monospecific antibody after modification of citrulline residues. J Invest Dermatol.

[21] Saiki M (2009). Recognition of the N-terminal histone H2A and H3 peptides by peptidylarginine deiminase IV. Protein Pept Lett.

[22] Wang Y, Wysocka J, Sayegh J, Lee YH, Perlin JR, Leonelli L, et al. Human PAD4 regulates histone arginine methylation levels via demethylimination. Science. 2004;306(5694):279–83. 10.1126/science.1101400 15345777

[23] van Boekel MA, Vossenaar ER, van den Hoogen FHJ, van Venrooij WJ. Autoantibody systems in rheumatoid arthritis: specificity, sensitivity and diagnostic value. Arthritis Res. 2002;4(2):87–93. 10.1186/ar395 PMC128920 11879544

[24] Kroot EJ, et al. The prognostic value of anti-cyclic citrullinated peptide antibody in patients with recent-onset rheumatoid arthritis. Arthritis Rheum. 2000;43(8):1831–5. 10.1002/1529-0131(200008)43:8<1831::AID-ANR19>3.0.CO;2-6 10943873

[25] Sohn DH, Rhodes C, Onuma K, Zhao X, Sharpe O, Gazitt T, et al. Local joint inflammation and histone citrullination in a murine model of the transition from preclinical autoimmunity to inflammatory arthritis. Arthritis Rheum. 2015;67(11):2877–87. 10.1002/art.39283 PMC4626401 26227989

[26] Khandpur R (2013). NETs are a source of citrullinated autoantigens and stimulate inflammatory responses in rheumatoid arthritis. Sci Transl Med.

[27] Liu CL, et al. Specific post-translational histone modifications of neutrophil extracellular traps as immunogens and potential targets of lupus autoantibodies. Arthritis Res Ther. 2012;14(1):R25. 10.1186/ar3707 PMC3392818 22300536

[28] Chapman EA (2019). Caught in a trap? Proteomic Analysis of Neutrophil Extracellular Traps in Rheumatoid Arthritis and Systemic Lupus Erythematosus. Front Immunol.

[29] Bennike TB, Carlsen TG, Ellingsen T, Bonderup OK, Glerup H, Bøgsted M, et al. Neutrophil extracellular traps in ulcerative colitis: a proteome analysis of intestinal biopsies. Inflamm Bowel Dis. 2015;21(9):2052–67. 10.1097/MIB.0000000000000460 PMC4603666 25993694

[30] Mastronardi FG (2006). Increased citrullination of histone H3 in multiple sclerosis brain and animal models of demyelination: a role for tumor necrosis factor-induced peptidylarginine deiminase 4 translocation. J Neurosci.

[31] Koch MW, Metz LM, Kovalchuk O. Epigenetic changes in patients with multiple sclerosis. Nat Rev Neurol. 2013;9(1):35–43. 10.1038/nrneurol.2012.226 23165337

[32] Ishida-Yamamoto A, Takahashi H, Iizuka H, Senshu T, Akiyama K, Nomura K. Decreased deiminated keratin K1 in psoriatic hyperproliferative epidermis. J Invest Dermatol. 2000;114(4):701–5. 10.1046/j.1523-1747.2000.00936.x 10733676

[33] Chumanevich AA (2011). Suppression of colitis in mice by cl-amidine: a novel peptidylarginine deiminase inhibitor. Am J Physiol Gastrointest Liver Physiol.

[34] Ishigami A, Ohsawa T, Hiratsuka M, Taguchi H, Kobayashi S, Saito Y, et al. Abnormal accumulation of citrullinated proteins catalyzed by peptidylarginine deiminase in hippocampal extracts from patients with Alzheimer's disease. J Neurosci Res. 2005;80(1):120–8. 10.1002/jnr.20431 15704193

[35] Vossenaar ER (2004). Expression and activity of citrullinating peptidylarginine deiminase enzymes in monocytes and macrophages. Ann Rheum Dis.

[36] Rogers GE, Harding HW, Llewellyn-Smith IJ. The origin of citrulline-containing proteins in the hair follicle and the chemical nature of trichohyalin, an intracellular precursor. Biochim Biophys Acta. 1977;495(1):159–75. 10.1016/0005-2795(77)90250-1 410454

[37] Ishigami A (2002). Human peptidylarginine deiminase type II: molecular cloning, gene organization, and expression in human skin. Arch Biochem Biophys.

[38] Chavanas S, Méchin MC, Takahara H, Kawada A, Nachat R, Serre G, et al. Comparative analysis of the mouse and human peptidylarginine deiminase gene clusters reveals highly conserved non-coding segments and a new human gene, PADI6. Gene. 2004;330:19–27. 10.1016/j.gene.2003.12.038 15087120

[39] Rus'd AA (1999). Molecular cloning of cDNAs of mouse peptidylarginine deiminase type I, type III and type IV, and the expression pattern of type I in mouse. Eur J Biochem.

[40] Guerrin M, et al. cDNA cloning, gene organization and expression analysis of human peptidylarginine deiminase type I. Biochem J. 2003;370(Pt 1):167–74. 10.1042/BJ20020870 PMC1223146 12416996

[41] Terakawa H, Takahara H, Sugawara K. Three types of mouse peptidylarginine deiminase: characterization and tissue distribution. J Biochem. 1991;110(4):661–6. 10.1093/oxfordjournals.jbchem.a123636 1778991

[42] Asaga H, Yamada M, Senshu T. Selective deimination of vimentin in calcium ionophore-induced apoptosis of mouse peritoneal macrophages. Biochem Biophys Res Commun. 1998;243(3):641–6. 10.1006/bbrc.1998.8148 9500980

[43] Deplus R (2014). Citrullination of DNMT3A by PADI4 regulates its stability and controls DNA methylation. Nucleic Acids Res.

[44] Lamensa JW, Moscarello MA (1993). Deimination of human myelin basic protein by a peptidylarginine deiminase from bovine brain. J Neurochem.

[45] Hsu PC (2014). Vimentin is involved in peptidylarginine deiminase 2-induced apoptosis of activated Jurkat cells. Mol Cell.

[46] Zhang X, Bolt M, Guertin MJ, Chen W, Zhang S, Cherrington BD, et al. Peptidylarginine deiminase 2-catalyzed histone H3 arginine 26 citrullination facilitates estrogen receptor α target gene activation. Proc Natl Acad Sci U S A. 2012;109(33):13331–6. 10.1073/pnas.1203280109 PMC3421185 22853951

[47] U, K.P (2014). Modulation of calcium-induced cell death in human neural stem cells by the novel peptidylarginine deiminase-AIF pathway. Biochim Biophys Acta.

[48] Asaga H (2001). Immunocytochemical localization of peptidylarginine deiminase in human eosinophils and neutrophils. J Leukoc Biol.

[49] Nakashima K, Hagiwara T, Yamada M (2002). Nuclear localization of peptidylarginine deiminase V and histone deimination in granulocytes. J Biol Chem.

[50] Arita K, Hashimoto H, Shimizu T, Nakashima K, Yamada M, Sato M. Structural basis for Ca (2+)-induced activation of human PAD4. Nat Struct Mol Biol. 2004;11(8):777–83. 10.1038/nsmb799 15247907

[51] Kan R (2012). Potential role for PADI-mediated histone citrullination in preimplantation development. BMC Dev Biol.

[52] Guo Q, Fast W. Citrullination of inhibitor of growth 4 (ING4) by peptidylarginine deminase 4 (PAD4) disrupts the interaction between ING4 and p53. J Biol Chem. 2011;286(19):17069–78. 10.1074/jbc.M111.230961 PMC3089551 21454715

[53] Lee YH (2005). Regulation of coactivator complex assembly and function by protein arginine methylation and demethylimination. Proc Natl Acad Sci U S A.

[54] Hagiwara T, Nakashima K, Hirano H, Senshu T, Yamada M. Deimination of arginine residues in nucleophosmin/B23 and histones in HL-60 granulocytes. Biochem Biophys Res Commun. 2002;290(3):979–83. 10.1006/bbrc.2001.6303 11798170

[55] Li P (2008). Regulation of p53 target gene expression by peptidylarginine deiminase 4. Mol Cell Biol.

[56] Tanikawa C, Espinosa M, Suzuki A, Masuda K, Yamamoto K, Tsuchiya E, et al. Regulation of histone modification and chromatin structure by the p53-PADI4 pathway. Nat Commun. 2012;3(1):676. 10.1038/ncomms1676 22334079

[57] Guo Q, Bedford MT, Fast W (2011). Discovery of peptidylarginine deiminase-4 substrates by protein array: antagonistic citrullination and methylation of human ribosomal protein S2. Mol BioSyst.

[58] Wright PW (2003). ePAD, an oocyte and early embryo-abundant peptidylarginine deiminase-like protein that localizes to egg cytoplasmic sheets. Dev Biol.

[59] Raijmakers R, Zendman AJW, Egberts WV, Vossenaar ER, Raats J, Soede-Huijbregts C, et al. Methylation of arginine residues interferes with citrullination by peptidylarginine deiminases in vitro. J Mol Biol. 2007;367(4):1118–29. 10.1016/j.jmb.2007.01.054 17303166

[60] Chavanas S (2006). Peptidylarginine deiminases and deimination in biology and pathology: relevance to skin homeostasis. J Dermatol Sci.

[61] Watanabe K (1988). Combined biochemical and immunochemical comparison of peptidylarginine deiminases present in various tissues. Biochim Biophys Acta.

[62] Darrah E (2012). Peptidylarginine deiminase 2, 3 and 4 have distinct specificities against cellular substrates: novel insights into autoantigen selection in rheumatoid arthritis. Ann Rheum Dis.

[63] Watanabe K, Senshu T. Isolation and characterization of cDNA clones encoding rat skeletal muscle peptidylarginine deiminase. J Biol Chem. 1989;264(26):15255–60. 2768262

[64] Chang X, Han J, Pang L, Zhao Y, Yang Y, Shen Z. Increased PADI4 expression in blood and tissues of patients with malignant tumors. BMC Cancer. 2009;9(1):40. 10.1186/1471-2407-9-40 PMC2637889 19183436

[65] Wang S, Wang Y. Peptidylarginine deiminases in citrullination, gene regulation, health and pathogenesis. Biochim Biophys Acta. 2013;1829(10):1126–35. 10.1016/j.bbagrm.2013.07.003 PMC3775966 23860259

[66] Kan R, Yurttas P, Kim B, Jin M, Wo L, Lee B, et al. Regulation of mouse oocyte microtubule and organelle dynamics by PADI6 and the cytoplasmic lattices. Dev Biol. 2011;350(2):311–22. 10.1016/j.ydbio.2010.11.033 PMC3031771 21147087

[67] Vossenaar ER, Zendman AJW, van Venrooij WJ, Pruijn GJM. PAD, a growing family of citrullinating enzymes: genes, features and involvement in disease. Bioessays. 2003;25(11):1106–18. 10.1002/bies.10357 14579251

[68] Arita K (2003). Crystallization and preliminary X-ray crystallographic analysis of human peptidylarginine deiminase V. Acta Crystallogr D Biol Crystallogr.

[69] Méchin MC (2007). Update on peptidylarginine deiminases and deimination in skin physiology and severe human diseases. Int J Cosmet Sci.

[70] Unno M (2012). Crystallization and preliminary X-ray crystallographic analysis of human peptidylarginine deiminase type III. Acta Crystallogr Sect F Struct Biol Cryst Commun.

[71] Knuckley B, Causey CP, Jones JE, Bhatia M, Dreyton CJ, Osborne TC, et al. Substrate specificity and kinetic studies of PADs 1, 3, and 4 identify potent and selective inhibitors of protein arginine deiminase 3. Biochemistry. 2010;49(23):4852–63. 10.1021/bi100363t PMC2884139 20469888

[72] Bicker KL (2012). Seeing citrulline: development of a phenylglyoxal-based probe to visualize protein citrullination. J Am Chem Soc.

[73] Nachat R (2005). Peptidylarginine deiminase isoforms 1-3 are expressed in the epidermis and involved in the deimination of K1 and filaggrin. J Invest Dermatol.

[74] Yang L, Tan D, Piao H (2016). Myelin basic protein Citrullination in multiple sclerosis: a potential therapeutic target for the pathology. Neurochem Res.

[75] Jang B, Jin JK, Jeon YC, Cho HJ, Ishigami A, Choi KC, et al. Involvement of peptidylarginine deiminase-mediated post-translational citrullination in pathogenesis of sporadic Creutzfeldt-Jakob disease. Acta Neuropathol. 2010;119(2):199–210. 10.1007/s00401-009-0625-x 20013286

[76] Krishnamurthy A (2019). Citrullination controls dendritic cell Transdifferentiation into osteoclasts. J Immunol.

[77] Nachat R, Méchin MC, Charveron M, Serre G, Constans J, Simon M. Peptidylarginine deiminase isoforms are differentially expressed in the anagen hair follicles and other human skin appendages. J Invest Dermatol. 2005;125(1):34–41. 10.1111/j.0022-202X.2005.23763.x 15982300

[78] Sipilä K (2014). Citrullination of collagen II affects integrin-mediated cell adhesion in a receptor-specific manner. FASEB J.

[79] Zhai Q (2017). Role of citrullination modification catalyzed by peptidylarginine deiminase 4 in gene transcriptional regulation. Acta Biochim Biophys Sin Shanghai.

[80] Zhang X (2016). Peptidylarginine deiminase 1-catalyzed histone citrullination is essential for early embryo development. Sci Rep.

[81] Meegan JE (2018). Citrullinated histone 3 causes endothelial barrier dysfunction. Biochem Biophys Res Commun.

[82] Young CH (2017). Citrullination regulates the expression of insulin-like growth factor-binding protein 1 (IGFBP1) in ovine uterine luminal epithelial cells. Reproduction.

[83] Golenberg N, et al. Citrullination regulates wound responses and tissue regeneration in zebrafish. J Cell Biol. 2020;219(4):e201908164. 10.1083/jcb.201908164 PMC7147109 32328635

[84] Sekeri-Pataryas, K.E. And T.G. Sourlingas, The differentiation-associated linker histone, H1.0, during the in vitro aging and senescence of human diploid fibroblasts*.* Ann N Y Acad Sci, 2007;1100:361–7. 10.1196/annals.1395.039 17460199

[85] Cherrington BD (2010). Potential role for peptidylarginine deiminase 2 (PAD2) in citrullination of canine mammary epithelial cell histones. PLoS One.

[86] Khan SA (2016). GnRH stimulates Peptidylarginine Deiminase catalyzed histone Citrullination in Gonadotrope cells. Mol Endocrinol.

[87] Lu X (2008). The effect of H3K79 dimethylation and H4K20 trimethylation on nucleosome and chromatin structure. Nat Struct Mol Biol.

[88] Xiao S (2017). SMARCAD1 contributes to the regulation of naive Pluripotency by interacting with histone Citrullination. Cell Rep.

[89] Li P, Li M, Lindberg MR, Kennett MJ, Xiong N, Wang Y. PAD4 is essential for antibacterial innate immunity mediated by neutrophil extracellular traps. J Exp Med. 2010;207(9):1853–62. 10.1084/jem.20100239 PMC2931169 20733033

[90] Wu Z, Deng Q, Pan B, Alam HB, Tian Y, Bhatti UF, et al. Inhibition of PAD2 improves survival in a mouse model of lethal LPS-induced Endotoxic shock. Inflammation. 2020;43(4):1436–45. 10.1007/s10753-020-01221-0 PMC7384922 32239392

[91] Zuo Y, et al. Neutrophil extracellular traps in COVID-19. JCI Insight. 2020;5(11). 10.1172/jci.insight.138999 PMC7308057 32329756

[92] Li P, Wang D, Yao H, Doret P, Hao G, Shen Q, et al. Coordination of PAD4 and HDAC2 in the regulation of p53-target gene expression. Oncogene. 2010;29(21):3153–62. 10.1038/onc.2010.51 PMC2913128 20190809

[93] Brown DT, Izard T, Misteli T (2006). Mapping the interaction surface of linker histone H1(0) with the nucleosome of native chromatin in vivo. Nat Struct Mol Biol.

[94] Li P, Hu J, Wang Y (2012). Methods for analyzing histone citrullination in chromatin structure and gene regulation. Methods Mol Biol.

[95] Leshner M (2012). PAD4 mediated histone hypercitrullination induces heterochromatin decondensation and chromatin unfolding to form neutrophil extracellular trap-like structures. Front Immunol.

[96] Buttinelli M (1999). The role of histone H1 in chromatin condensation and transcriptional repression. Genetica.

[97] Brinkmann V, Reichard U, Goosmann C, Fauler B, Uhlemann Y, Weiss DS, et al. Neutrophil extracellular traps kill bacteria. Science. 2004;303(5663):1532–5. 10.1126/science.1092385 15001782

[98] Cuthbert GL (2004). Histone deimination antagonizes arginine methylation. Cell.

[99] Clancy KW, Russell AM, Subramanian V, Nguyen H, Qian Y, Campbell RM, et al. Citrullination/methylation crosstalk on histone H3 regulates ER-target gene transcription. ACS Chem Biol. 2017;12(6):1691–702. 10.1021/acschembio.7b00241 PMC5536191 28485572

[100] Denis H (2009). Functional connection between deimination and deacetylation of histones. Mol Cell Biol.

[101] McNee G (2017). Citrullination of histone H3 drives IL-6 production by bone marrow mesenchymal stem cells in MGUS and multiple myeloma. Leukemia.

[102] Song G (2016). A novel PAD4/SOX4/PU.1 signaling pathway is involved in the committed differentiation of acute promyelocytic leukemia cells into granulocytic cells. Oncotarget.

[103] DeVore SB, et al. Histone citrullination represses MicroRNA expression, resulting in increased oncogene mRNAs in somatolactotrope cells. Mol Cell Biol. 2018;38(19):e00084-18. 10.1128/MCB.00084-18 PMC6146832 29987187

[104] Lu M, et al. Elevated histone H3 citrullination is associated with increased Beclin1 expression in HBV-related hepatocellular carcinoma. J Med Virol. 2020;92(8):1221-30. 10.1002/jmv.25663 31900950

[105] Li QQ (2017). Proteomic analysis of proteome and histone post-translational modifications in heat shock protein 90 inhibition-mediated bladder cancer therapeutics. Sci Rep.

[106] Wang L, Song G, Zhang X, Feng T, Pan J, Chen W, et al. PADI2-mediated Citrullination promotes prostate Cancer progression. Cancer Res. 2017;77(21):5755–68. 10.1158/0008-5472.CAN-17-0150 28819028

[107] Cherrington BD (2012). Potential role for PAD2 in gene regulation in breast cancer cells. PLoS One.

[108] Yao H, Li P, Venters BJ, Zheng S, Thompson PR, Pugh BF, et al. Histone Arg modifications and p53 regulate the expression of OKL38, a mediator of apoptosis. J Biol Chem. 2008;283(29):20060–8. 10.1074/jbc.M802940200 PMC2459274 18499678

[109] Qin H (2017). PAD1 promotes epithelial-mesenchymal transition and metastasis in triple-negative breast cancer cells by regulating MEK1-ERK1/2-MMP2 signaling. Cancer Lett.

[110] Stadler SC (2013). Dysregulation of PAD4-mediated citrullination of nuclear GSK3β activates TGF-β signaling and induces epithelial-to-mesenchymal transition in breast cancer cells. Proc Natl Acad Sci U S A.

[111] Duan Q, et al. Overexpression of PAD4 suppresses drug resistance of NSCLC cell lines to gefitinib through inhibiting Elk1-mediated epithelial-mesenchymal transition. Oncol Rep. 2016;36(1):551–8. 10.3892/or.2016.4780 27176594

[112] Sharma P (2019). Arginine Citrullination at the C-Terminal Domain Controls RNA Polymerase II Transcription. Mol Cell.

[113] Tohme S, Yazdani HO, al-Khafaji AB, Chidi AP, Loughran P, Mowen K, et al. Neutrophil extracellular traps promote the development and progression of liver metastases after surgical stress. Cancer Res. 2016;76(6):1367–80. 10.1158/0008-5472.CAN-15-1591 PMC4794393 26759232

[114] Qu Y, Olsen JR, Yuan X, Cheng PF, Levesque MP, Brokstad KA, Hoffman PS, Oyan AM, Zhang W, Kalland KH, Ke X (2018). Small molecule promotes β-catenin citrullination and inhibits Wnt signaling in cancer. Nat Chem Biol.

[115] Albrengues J, et al. Neutrophil extracellular traps produced during inflammation awaken dormant cancer cells in mice. Science. 2018;361(6409):eaao4227. 10.1126/science.aao4227 PMC6777850 30262472

[116] Yang LY, Luo Q, Lu L, Zhu WW, Sun HT, Wei R, et al. Increased neutrophil extracellular traps promote metastasis potential of hepatocellular carcinoma via provoking tumorous inflammatory response. J Hematol Oncol. 2020;13(1):3. 10.1186/s13045-019-0836-0 PMC6945602 31907001

[117] Tanikawa C (2009). Regulation of protein Citrullination through p53/PADI4 network in DNA damage response. Cancer Res.

[118] Brentville VA (2020). Post-translational modifications such as citrullination are excellent targets for cancer therapy. Semin Immunol.

[119] Boone BA (2015). The receptor for advanced glycation end products (RAGE) enhances autophagy and neutrophil extracellular traps in pancreatic cancer. Cancer Gene Ther.

[120] Song S (2019). A novel Citrullinated modification of histone 3 and its regulatory mechanisms related to IPO-38 antibody-labeled protein. Front Oncol.

[121] Zheng Y, Zhao G, Xu B, Liu C, Li C, Zhang X, et al. PADI4 has genetic susceptibility to gastric carcinoma and upregulates CXCR2, KRT14 and TNF-α expression levels. Oncotarget. 2016;7(38):62159–76. 10.18632/oncotarget.11398 PMC5308718 27556695

[122] Slack JL, Causey CP, Thompson PR (2011). Protein arginine deiminase 4: a target for an epigenetic cancer therapy. Cell Mol Life Sci.

[123] Hamam HJ, Palaniyar N. Post-translational modifications in NETosis and NETs-mediated diseases. Biomolecules. 2019;9(8):369. 10.3390/biom9080369 PMC6723044 31416265

[124] Wood DD (1996). Acute multiple sclerosis (Marburg type) is associated with developmentally immature myelin basic protein. Ann Neurol.

[125] Tranquill LR, Cao L, Ling NC, Kalbacher H, Martin RM, Whitaker JN. Enhanced T cell responsiveness to citrulline-containing myelin basic protein in multiple sclerosis patients. Mult Scler. 2000;6(4):220–5. 10.1177/135245850000600402 10962541

[126] Balint BL (2005). Arginine methylation provides epigenetic transcription memory for retinoid-induced differentiation in myeloid cells. Mol Cell Biol.

[127] Fuchs TA (2007). Novel cell death program leads to neutrophil extracellular traps. J Cell Biol.

[128] Papayannopoulos V, Metzler KD, Hakkim A, Zychlinsky A. Neutrophil elastase and myeloperoxidase regulate the formation of neutrophil extracellular traps. J Cell Biol. 2010;191(3):677–91. 10.1083/jcb.201006052 PMC3003309 20974816

[129] Barbu EA, Dominical VM, Mendelsohn L, Thein SL. Detection and quantification of histone H4 Citrullination in early NETosis with image flow Cytometry version 4. Front Immunol. 2020;11:1335. 10.3389/fimmu.2020.01335 PMC7378400 32765493

[130] Wang Y (2009). Histone hypercitrullination mediates chromatin decondensation and neutrophil extracellular trap formation. J Cell Biol.

[131] Berger-Achituv S (2013). A proposed role for neutrophil extracellular traps in cancer immunoediting. Front Immunol.

[132] Manjili MH (2017). Tumor dormancy and relapse: from a natural byproduct of evolution to a disease state. Cancer Res.

[133] Al-Khawashki MI (1979). Effects of althesin and its steroidal components on a variety of excised smooth muscle preparations. J Egypt Med Assoc.

[134] Demers M, Wong SL, Martinod K, Gallant M, Cabral JE, Wang Y, et al. Priming of neutrophils toward NETosis promotes tumor growth. Oncoimmunology. 2016;5(5):e1134073. 10.1080/2162402X.2015.1134073 PMC4910712 27467952

[135] Sase T (2017). Hypoxia-induced production of peptidylarginine deiminases and citrullinated proteins in malignant glioma cells. Biochem Biophys Res Commun.

[136] Redel J (1978). Chemical reactivity and biological properties of a series of aminochlorambucil derivatives. C R Acad Hebd Seances Acad Sci D.

[137] Cools-Lartigue J (2013). Neutrophil extracellular traps sequester circulating tumor cells and promote metastasis. J Clin Invest.

[138] Ho-Tin-Noé B (2009). Innate immune cells induce hemorrhage in tumors during thrombocytopenia. Am J Pathol.

[139] DuPre SA, Hunter KW (2007). Murine mammary carcinoma 4T1 induces a leukemoid reaction with splenomegaly: association with tumor-derived growth factors. Exp Mol Pathol.

[140] Demers M, Krause DS, Schatzberg D, Martinod K, Voorhees JR, Fuchs TA, et al. Cancers predispose neutrophils to release extracellular DNA traps that contribute to cancer-associated thrombosis. Proc Natl Acad Sci U S A. 2012;109(32):13076–81. 10.1073/pnas.1200419109 PMC3420209 22826226

[141] van der Windt DJ, Sud V, Zhang H, Varley PR, Goswami J, Yazdani HO, et al. Neutrophil extracellular traps promote inflammation and development of hepatocellular carcinoma in nonalcoholic steatohepatitis. Hepatology. 2018;68(4):1347–60. 10.1002/hep.29914 PMC6173613 29631332

[142] Jin W (2019). Tumor-infiltrating NETs predict postsurgical survival in patients with pancreatic ductal adenocarcinoma. Ann Surg Oncol.

[143] Saxena K, Jolly MK, Balamurugan K (2020). Hypoxia, partial EMT and collective migration: emerging culprits in metastasis. Transl Oncol.

[144] Thålin C, Lundström S, Seignez C, Daleskog M, Lundström A, Henriksson P, et al. Citrullinated histone H3 as a novel prognostic blood marker in patients with advanced cancer. PLoS One. 2018;13(1):e0191231. 10.1371/journal.pone.0191231 PMC5764486 29324871

[145] Mauracher LM (2018). Citrullinated histone H3, a biomarker of neutrophil extracellular trap formation, predicts the risk of venous thromboembolism in cancer patients. J Thromb Haemost.

[146] Tao L (2018). Polypharmacological profiles underlying the antitumor property of Salvia miltiorrhiza root (Danshen) interfering with NOX-dependent neutrophil extracellular traps. Oxidative Med Cell Longev.

[147] Hensen SM, Pruijn GJ. Methods for the detection of peptidylarginine deiminase (PAD) activity and protein citrullination. Mol Cell Proteomics. 2014;13(2):388–96. 10.1074/mcp.R113.033746 PMC3916641 24298040

[148] Mohanan S (2012). Potential role of peptidylarginine deiminase enzymes and protein citrullination in cancer pathogenesis. Biochem Res Int.

[149] Ulivi P, Mercatali L, Casoni GL, Scarpi E, Bucchi L, Silvestrini R, et al. Multiple marker detection in peripheral blood for NSCLC diagnosis. PLoS One. 2013;8(2):e57401. 10.1371/journal.pone.0057401 PMC3582604 23468981

[150] Yuzhalin AE, Gordon-Weeks AN, Tognoli ML, Jones K, Markelc B, Konietzny R, et al. Colorectal cancer liver metastatic growth depends on PAD4-driven citrullination of the extracellular matrix. Nat Commun. 2018;9(1):4783. 10.1038/s41467-018-07306-7 PMC6235861 30429478

[151] Chang X, Fang K (2010). PADI4 and tumourigenesis. Cancer Cell Int.

[152] Cantariño N, Musulén E, Valero V, Peinado MA, Perucho M, Moreno V, et al. Downregulation of the Deiminase PADI2 is an early event in colorectal carcinogenesis and indicates poor prognosis. Mol Cancer Res. 2016;14(9):841–8. 10.1158/1541-7786.MCR-16-0034 27280713

[153] Ju Z, Wang SY (2018). Prediction of citrullination sites by incorporating k-spaced amino acid pairs into Chou's general pseudo amino acid composition. Gene.

[154] Zhang Q (2017). Predicting Citrullination sites in protein sequences using mRMR method and random Forest algorithm. Comb Chem High Throughput Screen.

[155] Demers M, Wagner DD. NETosis: a new factor in tumor progression and cancer-associated thrombosis. Semin Thromb Hemost. 2014;40(3):277–83. 10.1055/s-0034-1370765 PMC4112728 24590420

[156] Cedervall J, Zhang Y, Olsson AK (2016). Tumor-induced NETosis as a risk factor for metastasis and organ failure. Cancer Res.

[157] Leffler J (2012). Neutrophil extracellular traps that are not degraded in systemic lupus erythematosus activate complement exacerbating the disease. J Immunol.

[158] Knuckley B (2010). Haloacetamidine-based inactivators of protein arginine deiminase 4 (PAD4): evidence that general acid catalysis promotes efficient inactivation. Chembiochem.

[159] Jones JE (2012). Synthesis and screening of a haloacetamidine containing library to identify PAD4 selective inhibitors. ACS Chem Biol.

[160] Teo CY (2017). Novel furan-containing peptide-based inhibitors of protein arginine deiminase type IV (PAD4). Chem Biol Drug Des.

[161] Witalison EE (2015). Molecular targeting of protein arginine deiminases to suppress colitis and prevent colon cancer. Oncotarget.

